# Targeted Immunotherapies for Cancers

**DOI:** 10.3390/cancers16010011

**Published:** 2023-12-19

**Authors:** Anthony Cheung, Alicia Chenoweth

**Affiliations:** 1 Breast Cancer Now Research Unit, School of Cancer & Pharmaceutical Sciences, King’s College London, Guy’s Cancer Centre, London SE1 9RT, UK; 2 St. John’s Institute of Dermatology, School of Basic & Medical Biosciences, King’s College London, Guy’s Hospital, London SE1 9RT, UK

Advancements in immunotherapy have revolutionized cancer treatment in a broad variety of hematological and solid malignancies and rejuvenated the field of cancer immunology. Several types of immunotherapies, including adoptive cell transfer and immune checkpoint inhibitors, have achieved durable clinical responses. This Special Issue highlights promising immune targeting strategies that may provide the basis for further translational investigations.

Nivolumab is an anti-PD-1 monoclonal antibody (mAb) which prolonged survival and retained the quality of life in a randomized, open-label phase III trial for patients with recurrent/metastatic squamous cell carcinoma of the head and neck (R/M SCCHN) [1]. It is now approved for use in the US, EU, and Canada for R/M SCCHN patients with progressive disease at or within six months after platinum-based therapy. Gogate et al. conducted a multinational retrospective study to capture the real-world utilization of nivolumab, demonstrating the effectiveness and safety of nivolumab in most of the patients and reporting stability or improvement in their health-related quality of life.

Despite the success in immunogenic tumors, due to the substantial heterogeneity of immune target expression and immune cell infiltration in tumor lesions, only subsets of cancer patients currently benefit from immunotherapy treatments [2,3]. This highlights an urgent unmet need for new immune targets and modification of mAbs for improved clinical function. Novel preclinical studies and clinical trials are investigating drugs targeting the immune checkpoint axis, either as monotherapy or in combination with other regimens [4]. Clinical results of immunotherapy in low immunogenic cancer types are poor, with response rates as low as 5% with monotherapy, especially in unselected patient populations [5,6]. A combination of chemotherapy or targeted therapy, together with checkpoint blockade, could demonstrate better success. Small-molecule drugs targeting CDK2 at efficacious doses may be associated with toxicity [7]. To address this limitation, Cheung et al. reported that in aggressive triple-negative breast cancers (TNBCs), CDK inhibition at suboptimal doses promotes immune cell recruitment to tumors and PD-L1 expression by surviving TNBC cells, and can complement checkpoint inhibitor immunotherapy. In orthotopic TNBC-bearing xenograft mice, suboptimal CDK inhibitor doses given sequentially ahead of dosing with the anti-PD-L1 antibody avelumab significantly restricted tumor growth compared with monotherapy [8].

Sato et al. reported another promising immune targeting strategy via adoptive cell therapy using chimeric antigen receptor engineered T cell (CAR-T) targeting carcinoembryonic antigen (CEA), a key target antigen which is highly expressed on the surface of pancreatic ductal adenocarcinoma (PDAC) cells [9]. They demonstrated that anti-CEA-CAR-T adoptive cell treatment induced regression of CEA-positive PDAC tumors in orthotopic mouse models, and that the CEA expression level was correlated with tumor heterogeneity and could be clinically used as a biomarker to select patients for anti-CEA-CAR-T therapy. Moreover, Christodoulou et al. gathered current data from the literature regarding the clinical testing and limitation of CAR-based therapies against difficult-to-treat acute myeloid leukemia. The authors discussed the potential benefits of using safer alternative lymphocytes, such as CAR-NK cells (natural killer cells with CARs) which has shown promise in preclinical studies [10,11].

Furthermore, a recombinant single-chain fragment variable (scFv) that contains the complete antigen-binding domains of a whole antibody has several advantages, such as stability and specificity against tumor antigens, with proven ability to penetrate tumor tissues and diffuse [12]. Muñoz-López et al. discussed the principle, generation, and applications of scFvs, particularly in the diagnosis and selective treatment of cancer. Breakthroughs in clinical trials have demonstrated that scFvs are safe molecules and present great antitumor activity when incorporated into CAR-T or bispecific T-cell engager (BiTE) systems.

Narbona et al. reported a new optimized version of colorectal cancer-targeted immunotoxin with low immunogenicity and enhanced antitumor activity in solid tumors. The immunogenic nature of bacterial and plant toxins represents a major drawback to their clinical use, as they can be recognized as foreign by the patient’s immune system and lead to the formation of anti-drug antibodies (ADAs) which neutralize and clear the immunotoxin, and can cause immune-related toxicities [13,14]. The authors designed and characterized two different variants of non-immunogenic immunotoxins based on a de-immunized variant of the ribotoxin α-sarcin, one lacking CD4+ T-cell epitopes and one included a recognition and cleavage furin linker to improve the toxin release to the cytosol. The data showed efficient antitumor effects both in vitro and in vivo.

In summary, this Special Issue of *Cancers* is a collection of articles discussing several immune targeting strategies, including the combination of cell cycle inhibitors in harmony with checkpoint inhibitor immunotherapy, chimeric antigen receptor engineered T or NK cell therapies, scFvs, and optimized immunotoxins. These may provide the basis for further translational investigations, particularly for patients who do not adequately benefit from currently available therapies.

In total, six papers were accepted for publication and inclusion in this Special Issue (comprising four original articles and two reviews).

## References

[1] Ferris R.L., Blumenschein G., Fayette J., Guigay J., Colevas A.D., Licitra L., Harrington K., Kasper S., Vokes E.E., Even C. (2016). Nivolumab for Recurrent Squamous-Cell Carcinoma of the Head and Neck. N. Engl. J. Med..

[2] Pitt J.M., Vetizou M., Daillere R., Roberti M.P., Yamazaki T., Routy B., Lepage P., Boneca I.G., Chamaillard M., Kroemer G. (2016). Resistance Mechanisms to Immune-Checkpoint Blockade in Cancer: Tumor-Intrinsic and -Extrinsic Factors. Immunity.

[3] O’Donnell J.S., Teng M.W.L., Smyth M.J. (2019). Cancer immunoediting and resistance to T cell-based immunotherapy. Nat. Rev. Clin. Oncol..

[4] Kaplon H., Crescioli S., Chenoweth A., Visweswaraiah J., Reichert J.M. (2023). Antibodies to watch in 2023. MAbs.

[5] Adams S., Schmid P., Rugo H.S., Winer E.P., Loirat D., Awada A., Cescon D.W., Iwata H., Campone M., Nanda R. (2019). Pembrolizumab monotherapy for previously treated metastatic triple-negative breast cancer: Cohort A of the phase II KEYNOTE-086 study. Ann. Oncol..

[6] Dirix L.Y., Takacs I., Jerusalem G., Nikolinakos P., Arkenau H.T., Forero-Torres A., Boccia R., Lippman M.E., Somer R., Smakal M. (2018). Avelumab, an anti-PD-L1 antibody, in patients with locally advanced or metastatic breast cancer: A phase 1b JAVELIN Solid Tumor study. Breast Cancer Res. Treat..

[7] Merrick K.A., Wohlbold L., Zhang C., Allen J.J., Horiuchi D., Huskey N.E., Goga A., Shokat K.M., Fisher R.P. (2011). Switching Cdk2 on or off with small molecules to reveal requirements in human cell proliferation. Mol. Cell.

[8] Cheung A., Chenoweth A.M., Quist J., Sow H.S., Malaktou C., Ferro R., Hoffmann R.M., Osborn G., Sachouli E., French E. (2022). CDK Inhibition Primes for Anti-PD-L1 Treatment in Triple-Negative Breast Cancer Models. Cancers.

[9] Gansauge S., Gansauge F., Beger H.G. (1996). Molecular oncology in pancreatic cancer. J. Mol. Med..

[10] Caruso S., De Angelis B., Del Bufalo F., Ciccone R., Donsante S., Volpe G., Manni S., Guercio M., Pezzella M., Iaffaldano L. (2022). Safe and effective off-the-shelf immunotherapy based on CAR.CD123-NK cells for the treatment of acute myeloid leukaemia. J. Hematol. Oncol..

[11] Albinger N., Pfeifer R., Nitsche M., Mertlitz S., Campe J., Stein K., Kreyenberg H., Schubert R., Quadflieg M., Schneider D. (2022). Primary CD33-targeting CAR-NK cells for the treatment of acute myeloid leukemia. Blood Cancer J..

[12] Munoz-Lopez P., Ribas-Aparicio R.M., Becerra-Baez E.I., Fraga-Perez K., Flores-Martinez L.F., Mateos-Chavez A.A., Luria-Perez R. (2022). Single-Chain Fragment Variable: Recent Progress in Cancer Diagnosis and Therapy. Cancers.

[13] Sauna Z.E., Lagasse D., Pedras-Vasconcelos J., Golding B., Rosenberg A.S. (2018). Evaluating and Mitigating the Immunogenicity of Therapeutic Proteins. Trends Biotechnol..

[14] Mazor R., King E.M., Pastan I. (2018). Strategies to Reduce the Immunogenicity of Recombinant Immunotoxins. Am. J. Pathol..

